# Characteristics of Self-Harm in an Emergency Situation Among Youth: A Longitudinal Five- Year Cohort Study

**DOI:** 10.1007/s10802-025-01309-x

**Published:** 2025-04-26

**Authors:** Cristina Varo, Maria Luisa Barrigón, Julia Rider, Pablo Reguera, Ana Mayo-Jaraquemada, Manuel Canal-Rivero, Nathalia Garrido-Torres, Enrique Baca-Garcia, Miguel Ruiz-Veguilla, Benedicto Crespo-Facorro

**Affiliations:** 1 Department of Personality, Assessment, and Psychological Treatments, Universidad de Sevilla, Seville, 41018 Spain; 2 Institute of Psychiatry and Mental Health,, Hospital General Universitario Gregorio Marañón, IiSGM, School of Medicine, Universidad Complutense, Madrid, Spain; 3 Mental Health Unit, Virgen del Rocio University Hospital, Seville, Spain; 4 Translational Psychiatry Group, Seville Biomedical Research Institute (IBIS), CSIC, Seville, Spain; 5 Spanish Network for Research in Mental Health (CIBERSAM), Madrid, Spain; 6 Department of Psychiatry, University of Seville, Seville, Spain; 7 Department of Psychiatry, Fundación Jiménez Díaz Hospital, Madrid, Spain; 8 Department of Psychiatry, Autónoma University, Madrid, Spain; 9 Department of Psychiatry, Rey Juan Carlos University Hospital, Móstoles, Spain; 10 Department of Psychiatry, General Hospital of Villalba, Madrid, Spain; 11 Department of Psychiatry, Infanta Elena University Hospital, Valdemoro, Spain; 12 Universidad Católica del Maule, Talca, Chile; 13 Department of Psychiatry, University Hospital Virgen del Rocio, IBIS-CSIC, University of Seville, CIBERSAM G26, IBiS, Avd. Manuel Siurot s/n, Seville, 41013 Spain

**Keywords:** Self-harm, COVID-19 pandemic, Young adults, Risk factors, Mental health

## Abstract

Self-harm is a growing phenomenon among young people. This study examined self-harm rates in youths over five years, before and after the onset of the COVID-19 pandemic. Additionally, we explored risk factors influencing self-harm repetition. We conducted a retrospective analysis of people under 25 years who presented with self-harm at an emergency department in Seville, Spain, from January 1st 2018 to December 31st 2022. Participants were identified through hospital records. We used Joinpoint regression analysis to assess changes in trends and compared demographic and clinical variables between pre- and post-pandemic periods. Cox regression analysis was employed to identify predictors of repeated self-harm. The study included of 726 individuals, with 282 (38.84%) and 444 (61.16%) first-time self-harm cases before and after the COVID-19 pandemic onset, respectively. A significant increase in self-harm rates was observed from December 2020 to March 2021. Post-pandemic, there was an over-representation of 10-14-year-olds, increased substance use rates, and a higher proportion of suicide attempts compared to non-suicidal self-injuries. Affective disorders, personality disorders, lifetime psychiatric history, and previous non-suicidal self-injuries were associated with higher rates of recurring self-harm episodes. Self-harm among young people increased one year after the initial COVID-19 outbreak. Post-pandemic first-time self-harm cases exhibited higher rates of substance use and tended to engage in suicide attempts, particularly among 10-14-year-olds. Recurrence was associated with psychiatric diagnosis, psychiatric history and non-suicidal self-harm history.

**Supplementary Information:**

The online version contains supplementary material available at 10.1007/s10802-025-01309-x.

## Introduction

Suicide is a global public health issue requiring urgent attention. In 2019, over 700,000 deaths were attributed to suicide, with a global age-standardized rate of 9.0 per 100,000 people (Knipe et al., 2022). Suicide impacted all age groups, being the fourth leading cause of death among 15–29-year-olds. Moreover, in numerous countries, it is the primary or second leading cause of death among adolescents and young adults (World Health Organization [WHO], 2021).

Self-harm, defined as any act of self-injury, regardless of intent (Knipe et al., 2022; Skegg, 2005) and future suicide risk are strongly associated (de la Torre-Luque et al., 2023; Hawton et al., 2012a, b). It is estimated that approximately 20 self-harm episodes occur for every suicide death each year. In particular, among adolescents and young adults, self-harm increases the risk of death by suicide by approximately tenfold (Hawton et al., 2003).

Adolescence and young adulthood are critical developmental stages characterized by significant physical, emotional, and social changes. During these periods, individuals face unique stressors such as academic pressures, peer relationships, and identity formation, which can contribute to heightened emotional distress (National Academies of Sciences et al., 2019). This developmental context is crucial for understanding why adolescents and young adults may be particularly vulnerable to self-harming behaviors. Research indicates that psychological factors such as prior self-harm tendencies and psychiatric illnesses intersect with psychosocial influences like strained family dynamics and substance use to elevate the risk of self-harm repetition in this demographic (Carballo et al., 2020).

Prior to the pandemic, a steady increase in lifetime self-harm rates among individuals aged 16–24 were observed between 2000 and 2014 (McManus & Gunnell, 2020). The COVID-19 pandemic subsequently amplified this trend, emerging as a risk factor for self-harm (Farooq et al., 2021; Samji et al., 2022). The interpersonal-psychological theory of suicide provides a framework for understanding these increased risks. This theory posits that three main factors contribute to suicide risk: thwarted belongingness, perceived burdensomeness, and acquired capability for suicide (Van Orden et al., 2010). Public health measures implemented to reduce virus spread, such as physical distancing, social and physical isolation, wearing face masks or travel restrictions have exacerbated these factors (Farooq et al., 2021; Samji et al., 2022). The consequences of the effect of these pandemic-response measures and the exposure to the COVID-19 infection itself had a negative impact on population mental health. Individuals have experienced isolation (potentially increasing thwarted belongingness), loss, fear of infection, anxiety, disruption of routine, limited access to mental health support, economic insecurity (potentially increasing perceived burdensomeness), and uncertainty about the future. The pandemic may have also increased capability for suicide through repeated exposure to loss, economic hardship, social changes, and fear of COVID-19, potentially leading to emotional desensitization and reduced fear of death.

Understanding the factors influencing self-harm repetition, particularly in the context of the COVID-19 pandemic, is crucial for developing effective prevention strategies. Research has identified various risk factors for self-harm in adolescents and young adults, including psychological (e.g., psychiatric illness, prior self-harm), psychosocial (e.g., alcohol abuse, strained relationships), and sociodemographic factors (e.g., age, gender, ethnicity) (Rahman et al., 2021). Although while these risk factors for self-harm such as depression, alcohol misuse, and gender, often overlap with those for repeated self-harm (Rahman et al., 2021), the risk of repetition requires specific attention. Past self-harm experiences predict future tendencies (Plener et al., 2015), with 15–25% of adolescents repeating self-harm within a year (Hawton et al., 2012). However, research on repeated self-harm in young people remains limited, resulting in a lack of understanding of what distinguishes those who repeat from those who do not.

The COVID-19 pandemic has created a particularly vulnerable scenario for individuals with a history of self-harm. As a significant risk factor for the development, relapse or exacerbation of mental health conditions, the pandemic may further strain coping mechanisms, potentially increasing the risk of self-harm repetition. This unique context underscores the importance of investigating the interplay between pandemic-related stressors and existing risk factors for self-harm repetition. A better understanding of of these complex interactions will facilitate the development of tailored and effective suicide prevention strategies (Fedyszyn et al., 2016).

Despite the observed increase in self-harm rates among adolescents and young adults both during and after the COVID-19 pandemic, the specific characteristics of self-harm reattempts among at-risk individuals remain unclear. A comprehensive analysis of how self-harm patterns have evolved post-pandemic is crucial for informing evidence-based mental health policies and developing targeted prevention and management strategies. However, most existing studies examining self-harm in psychiatric emergency departments have limited their follow-up to 2021, leaving a critical gap in our understanding of longer-term pandemic effects. We address this limitation by extending the analysis through December 2022, providing a more comprehensive view of self-harm trends and characteristics in the post-pandemic period.

This study aimed to investigate changes in self-harm and characteristics among individuals aged 10–25 years following the COVID-19 pandemic compared to pre-pandemic times. Additionally, we explored the role of demographic, clinical, and episode-related factors in predicting self-harm repetition. To achieve these objectives, we conducted a comprehensive longitudinal study analyzing medical records from January 2018 to December 2022 at a major hospital in Seville, Spain.

## Methods

### Study Design and Setting

This retrospective study analyzed medical records of people under 25 years of age who were attended after a self-harm episode in the emergency department of University Hospital Virgen del Rocio (UHVR) at Seville (South-western Spain) from January 1st, 2018, to December 31st 2022.

HUVR provides tax-funded medical care to approximately 800,000 inhabitants in the province of Seville, encompassing three of the five health districts within the province. Geographically, it serves two distinct areas: an urban zone comprising two health districts (Seville and Southern Sevilla), and a metropolitan area (Aljarafe).

### Participants

Participants included all individuals under 25 years of age who presented to the UHVR emergency department with a self-harm episode during the study period.

### Measures and Procedures

#### Self-harm Episodes

*Self-harm* was defined as a non-fatal deliberate action of self-poisoning and/or self-injury, regardless of the underlying motives (Knipe et al., 2022; Skegg, 2005). Episodes were categorized either as suicide attempts or non-suicidal self-injuries, based on the intention behind the act. We considered self-harm episodes with the intention to die as suicide attempts (O’Carroll et al., 1996; Silverman et al., 2007) and injuries deliberately inflicted upon oneself without the intention to die as *non-suicidal self-injury* (NSSI) (De Leo et al., 2021).

The categorization was determined by clinical assessment during the emergency department visit and recorded in the medical records and in a dedicated register. After the implementation of the Suicide Prevention Protocol (Crespo-Facorro et al., 2021) at the Mental Health Department of UHVR in 2018, information on self-harm episodes attended to in the emergency room was collected on a daily basis and categorized according to an ad hoc risk classification system, based on international criteria (O’Carroll et al., 1996; Posner et al., 2007; Silverman et al., 2007). All clinicians involved in the management of psychiatric emergencies underwent training in this classification system.

#### Clinical Variables

The following information was extracted from clinical records by one research assistant (JR) for al index episodes: (1) demographic variables: age and gender; (2) clinical variables: lifetime substance use (yes/no), psychiatric diagnosis at discharge (categorized according to the International Classification of Diseases, Tenth Revision (ICD-10) as follows: affective disorders (F30–F39), anxiety disorders (F40–F48), personality disorders (F60–F69), and other disorders (remaining F codes), and lifetime psychiatric history (yes/no); and (3) characteristics related to the episode: date, type of episode (attempted suicide or NSSI), the method(s) of self-harm coded according to ICD-10 codes for intentional injury (X60–X84), consumption of drugs and/or alcohol at the time of the episode (yes/no), hospitalization (medical or psychiatric yard) after the episode, and lifetime history of NSSI (yes/no).

#### COVID-19 Timeframe

The date of the episode was registered and contextualized within the COVID-19 pandemic timeframe in Spain. We set the start of the pandemic on March 13th 2020, when national emergency and lockdown was declared (Royal Decree 463/2020 of the Spanish State Official Bulletin; BOE-A-2020-3692) and divided our sample into those episodes taking place during the “pre-COVID-19 period” (January 2018 - March 13th 2020) and those taking place during the “post-COVID-19 period” (March 14th 2020– December 31st 2022).

#### Recurrence

We identified recurring self-harm episodes of a full five-year follow-up and collected the following information: total number of repeated episodes within one month after the index episode, psychiatric hospitalization (yes/no), and COVID-19 period (pre or post-pandemic).

#### Data Extraction Procedure

The research assistant, who had previously undergone training in the current suicide protocol used at the hospital, undertook a review of the records of self-harm episodes. This entailed the extraction of information on variables previously described and their placement in a pseudo-anonymous spreadsheet.

Complex or borderline cases were assessed by senior clinicians (MRV and MLB) with experience in suicide risk assessment. The research team employed a consensus approach to reach a decision, engaging in discussion to ascertain the most accurate categorization.

### Statistical Analysis

Data analysis involved four stages. Firstly, to analyze temporal trends and identify changes in new self-harm episodes per month “pre-COVID-19 period” and “post-COVID-19 period”, we conducted a joinpoint regression analysis on the log-transformed monthly counts was conducted using Joinpoint software version 4.9.0.0 (Fernández-Navarro et al., 2016; Kim et al., 2000). We considered a maximum of three joinpoints, as recommended for time series with limited monthly observations, to balance model complexity and interpretability. The final model was selected using permutation tests to identify the most parsimonious solution, based on the lowest permutation p-value (*p* <.05). These regression models determined the optimal number of joinpoints corresponding to significant changes in the slope of monthly counts over time. We estimated crude incidence rate ratios and 95% confidence intervals by comparing the monthly incidence rate of 2018 with the corresponding month in 2022. Based on the starting and ending months of each segment identified by joinpoints, we calculated the monthly percent change (MPC) and 95% confidence interval. Models were fitted using least squares regression on log-transformed data to estimate relative changes in incidence over time, ensuring appropriate capture of trends.

Secondly, descriptive analyses were conducted to compare sociodemographic and clinical characteristics of adolescents/young adults with a first self-harm episode between “Pre-COVID-19 period” and “Post-COVID-19 period” using t-tests for the continuous variables and Chi-square tests for the categorical variables. Also effect sizes (Cramer’s V) were calculated to estimate the magnitude of the differences between both groups (0.1 = small, 0.3 = medium, 0.5 or greater = large) (Cohen, 1992).

Thirdly, univariable Cox proportional hazards regression models examined the following 10 variables as potential predictors of repeated suicide attempts measured over five years of follow-up: age group at index episode, gender, diagnosis, lifetime psychiatric history, lifetime history of non-suicidal self-injuries, type of index, self-harm method of index episode, psychiatric and somatic hospitalization after index episode, and COVID-19 period. Only those covariables with *p* values *p* <.05 were then entered into a multivariable Cox regression analysis. Stepwise backward selection was used to minimize the risk of detection of a true effect (type II error). For individuals with multiple self-harm episodes, only the first repeat episode was analyzed. Statistical significance was set at *p* values *p* <.05. All analyses were performed with Statistical Package for Social Sciences software version 23 (SPSS Inc., Chicago, IL, USA).

### Ethical Issues

The study was approved by the Local Ethics Committee - the Research Ethics Committee of the University Hospitals Virgen Macarena-Virgen del Rocío - and no informed consent from the participants was required given the nature of the study. One research assistant, bound by a confidentiality agreement, was responsible for data extraction from medical records. During this process, all patient information was pseudonymized and patient identifiers were replaced with unique study codes in an Excel spreadsheet, effectively anonymizing the data for analysis. Access to the pseudonymized dataset was restricted to authorized research personnel and all data analysis was conducted using the pseudonymized dataset, thus further protecting patient privacy.

## Results

### Characteristics of Self-harm Episodes According to COVID-19 Period

**Fig. 1:**
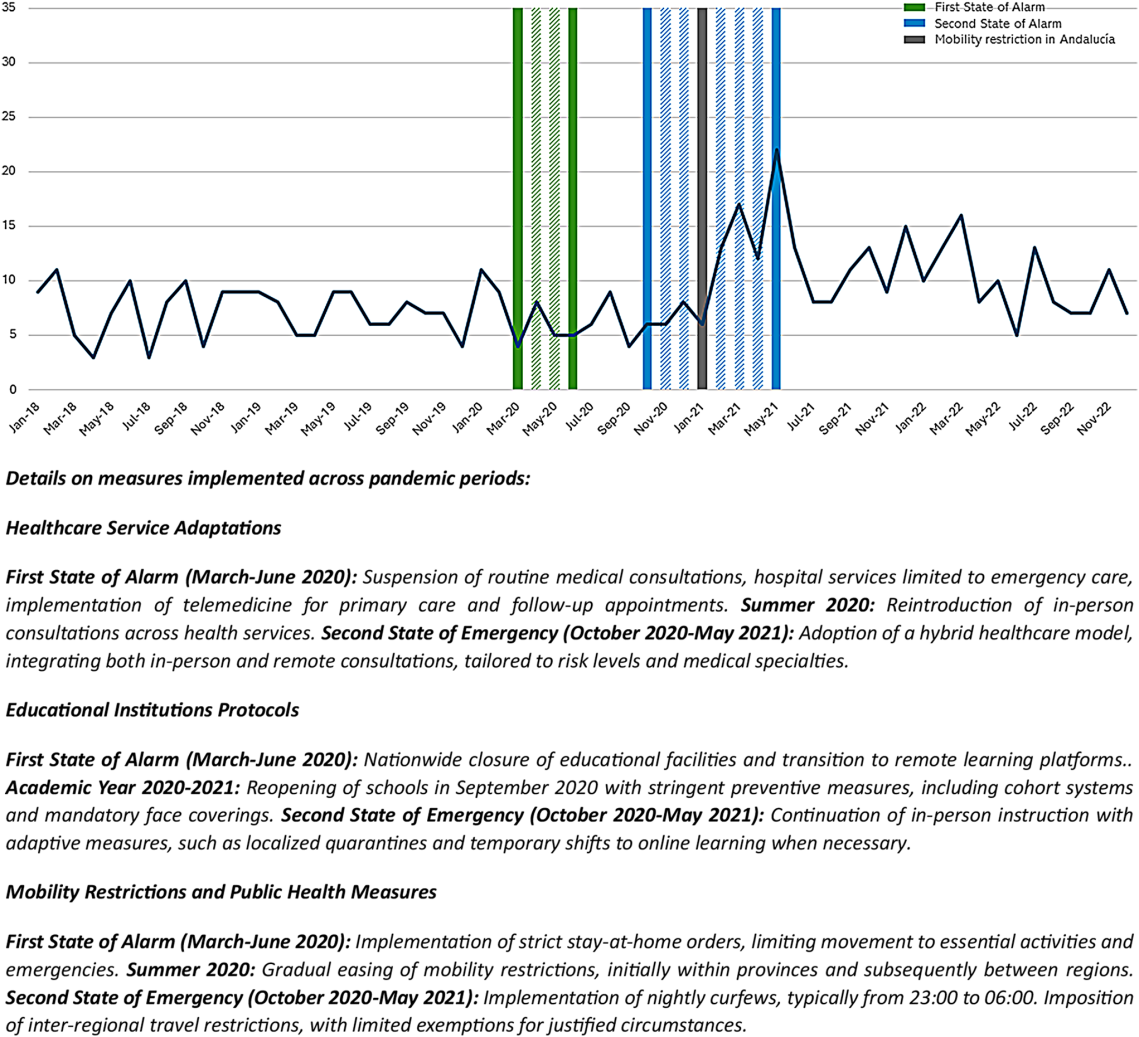
Trend in self-harm episodes in the emergency department in adolescents and young adults from 2018 to 2022

**Fig. 2:**
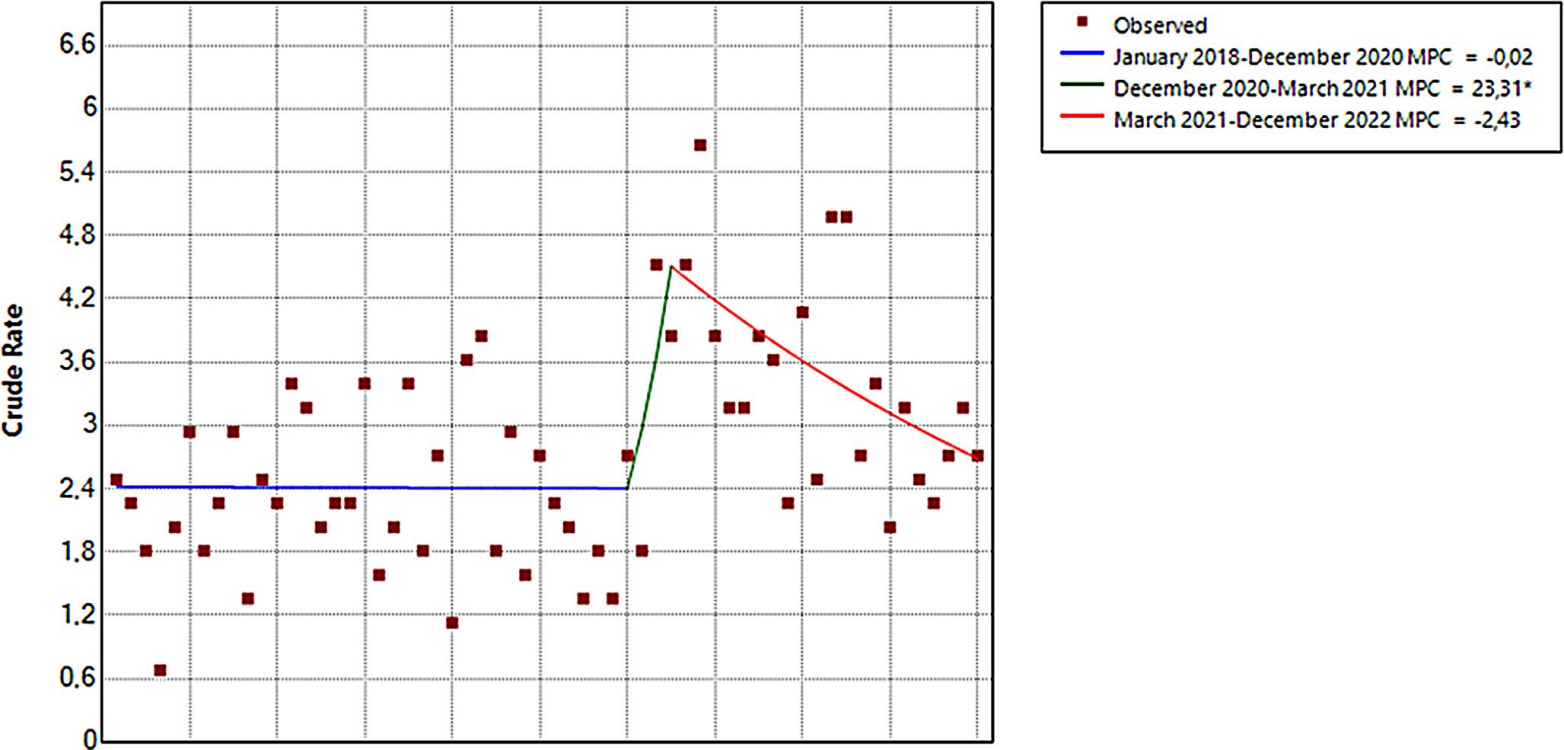
Joinpoint analysis

Between January 2018 and December 2022, 726 self-harm episodes were attended in patients under 25 years of age, 282 (38.84%) during the “pre-COVID-19 period” and 444 (61.16%) during the “post-COVID-19 period”. Notably, out of the 726 cases, 659 (90.77%) had their first registered self-harm episode during the 2018–2022 period. Most frequent method was self-poisoning in both pre and post-pandemic periods, followed by cutting or piercing (Table 1). Figure 1 shows the representation of attendances due to self-harms in adolescents and young adults during the 2018–2022 period. Figure 2 shows the whole series of first episodes with results from the joinpoint regression. Two joinpoints were identified: December 2020 and March 2021. Then, there was a significant increase of 23.31% in first self-harm episodes from December 2020 to March 2021 [MPC = 23.31 (95% *CI* = 0.22, 35.68)], followed by a significant decrease of 2.43% from March 2021 to December 2022 [MPC = -2.43 (95% *CI* = -6.06, 0.58)] (for more information on the calculation of Join-point see Supplementary Materials).

Episodes in the different COVID-19 periods differed in age, lifetime substance use and type of event. Individuals aged 10–14 years were over-represented in “post-COVID-19 period” (*χ²* = 6.86, *p* = .032). Similarly, in comparison with “pre-COVID-19 period”, in “post-COVID-19 period”, the rate of lifetime substance use was higher (*χ²* = 4.40, *p* = .036), and suicide attempt was more frequent than non-suicidal self-injuries (*χ²* = 3.91, *p* = .048). Detailed information regarding all remaining variables at the index episode during COVID-19 periods is shown in Table 1.

**Table 1:** Demographic and clinical characteristics of index episode according to COVID-19 period

		Pre-COVID-19 period* (*n* = 282) *N*(%)	Post-COVID-19 period* (*n* = 444) *N*(%)	X^2^	*p*-value	Cramer’s V (df)
**Age group at index episode**				6.86	**0.032**	0.10 (2)
10–14 years old		40 (14.23)	98 (22.10)			
15–19 years old		142 (50.53)	205 (46.20)			
20–26 years old		99 (35.23)	141 (31.80)			
**Gender (male)**		89 (31.60)	128 (28.82)	0.61	0.433	0.03 (1)
**Lifetime substance use (yes)**		58 (21.00)	118 (28.10)	4.40	**0.036**	0.08 (1)
**Diagnosis**				7.94	0.094	0.11 (1)
None		107 (37.94)	165 (37.20)			
Affective disorder		34 (12.10)	50 (11.30)			
Anxiety or adjustment disorder		55 (19.50)	118 (26.60)			
Personality Disorder		40 (14.20)	40 (9.00)			
Other		43 (16.31)	71 (16.00)			
**Lifetime psychiatric history (yes)**		195 (69.14)	281 (63.30)	2.37	0.123	0.11 (4)
**Lifetime non-suicidal self-injury (yes)**		125 (45.00)	186 (42.80)	0.33	0.562	0.02 (1)
**Type of index episode**				3.91	**0.048**	0.07 (1)
Suicide attempt		198 (70.21)	341 (76.80)			
Non suicidal self-injury		84 (29.80)	103 (23.20)			
**Method of index episode**				6.31	0.177	0.09 (4)
Hanging, strangling, suffocation		6 (2.12)	12 (2.72)			
Cutting or piercing		68 (24.11)	91 (20.63)			
Self-poisoning		184 (65.24)	303 (68.70)			
Jumping from height		5 (1.80)	17 (3.90)			
Other		19 (6.73)	18 (4.10)			
**Consumption of drugs or alcohol at the time of the act (yes)**		25 (9.70)	33 (8.52)	0.26	0.613	0.02 (1)
**Somatic hospitalization (yes)**		76 (27.94)	97 (22.50)	2.65	0.103	0.06 (1)
**Psychiatric hospitalization (yes)**		55 (19.90)	71 (16.32)	1.45	0.228	0.05 (1)
**Recurring self-harm episodes**		122 (43.31)	129 (29.10)		0.613	0.09 (1)
**Recurring self-harm episodes within one month of the index episode**		34 (27.90)	47(36.40)		**< 0.001**	0.15 (1)

### Characteristics of Recurring Self-Harm Episodes Over a Five-year Follow-up Period

Out of all the individuals involved in their first registered self-harm episodes between January 2018 and December 2022, 251 individuals (34.57%) repeated it after the initial event. Of these, 182 were women (72.50%), while 69 were men (27.50%). Almost half of them were between the ages of 15 and 19 years (*n* = 124). Most of these individuals had a psychiatric diagnosis (74.10%, *n* = 186), previous history of mental health issues (79%, *n* = 200), and a history of non-suicidal self-injuries (61%, *n* = 153). The most common methods of self-harm were self-poisoning (57.40%, *n* = 144) and cutting or piercing (32.30%, *n* = 32.3). More than two-thirds did not require psychiatric hospitalization after the index episode (77.30%, *n* = 194) (Table 2). After conducting the post-hoc analysis, no significant differences were found in recurring episodes as a function of COVID-19 periods (*n* = 122, 43.31% vs. *n* = 129, 29.10%; *p* = .613). However, upon further examination of recurring episodes within less than 30 days, a larger number of individuals made a new self-harm act in the “post-COVID-19 period” (*n* = 46, 36.40% vs. *n* = 34, 27.90%, *p* <.001).

**Table 2:** Cox proportional hazard ratios (HRs) survival analysis for recurring self-harm episodes

Variable	Repeated Suicide Attempt. *n* (%)	Univariable HR (95% CI)	*p*-value	Multivariable HR (95% CI)	*p*-value
Age group at index episode					
10–14 years old	45 (17.92)	1			
15–19 years old	124 (49.40)	0.97 (0.67–1.39)	0.869		
20–26 years old	82 (32.70)	1.00 (0.76–1.32)	0.992		
Gender					
Female	182 (72.50)	1			
Male	69 (27.50)	0.86 (0.65–1.14)	0.286		
Psychiatric Diagnosis					
None	65 (25.90)	1		1	
Affective disorder	36 (14.34)	2.04 (1.36–3.07)	**0.001**	1.54 (1.01–2.34)	**0.045**
Anxiety or adjustment disorder	56 (22.31)	1.47 (1.03–2.11)	**0.034**	1.2 (0.83–1.74)	0.338
Personality disorder	46 (18.32)	3.19 (2.19–4.67)	**< 0.001**	2.06 (1.38–3.08)	**< 0.001**
Others	48 (19.12)	1.84 (1.27–2.68)	**0.001**	1.34 (0.91–1.99)	0.141
Lifetime psychiatric history					
No	51 (20.31)	1			
Yes	200 (79.70)	2.44 (1.79–3.32)	**< 0.001**	1.77 (1.27–2.47)	**< 0.001**
Lifetime non-suicidal self-injury					
No	98 (39.00)	1			
Yes	153 (61.00)	2.49 (0.31–0.52)	**< 0.001**	1.79 (1.36–2.37)	**< 0.001**
Type of index episode					
Suicide attempt	170 (67.72)	1			
Non suicidal self-injury	81 (32.30)	1.52 (1.17–1.98)	**0.002**	1.07 (0.78–1.46)	0.656
Method of index self-harm episode					
Hanging, strangling, suffocation	5 (1.90)	1		1	
Cutting or piercing	81 (32.30)	3.11 (1.14–8.49)	**0.027**	2.57 (0.94–7.06)	**0.066**
Self-poisoning	144 (57.40)	1.53 (0.57–4.12)	0.404	1.63 (0.60–4.42)	0.334
Jumping from height	6 (2.40)	1.63 (0.46–5.78)	0.448	1.53 (0.43–5.44)	0.509
Other	15 (6.01)	2.21 (0.73–6.67)	0.158	2.14 (0.71–6.49)	0.179
Psychiatric hospitalization					
No	194 (77.30)	1			
Yes	49 (19.52)	1.19 (0.87–1.64)	0.265		
COVID-19 period					
Pre-COVID-19 period	122 (48.60)	1			
Post-COVID-19 period	129 (51.40)	1.07 (0.83–1.39)	0.595		

Footnote: *Pre-COVID-19 period (2018- Mar 13, 2020); Post-COVID-19 period (Mar 14, 2020-Dec 31, 2022)

### Predictors of Recurring Self-Harm Episodes

**Fig. 3:**
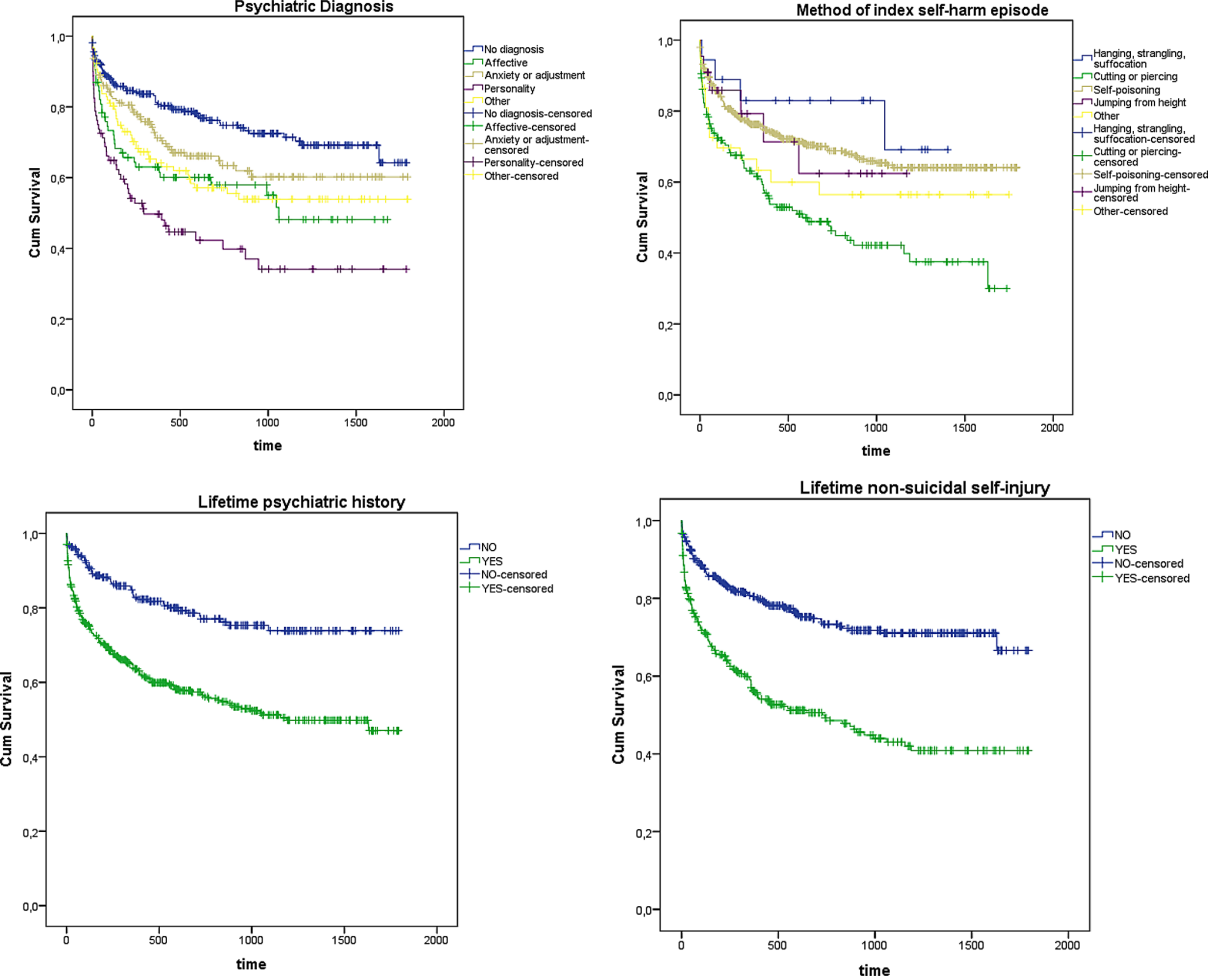
Survival functions of recurring self-harm episodes after the index episode according to each significant multivariable predictor

Table 2 shows univariable and multivariable hazard ratios for the potential predictors of recurring self-harm episodes. In the multivariate analysis, certain covariates presented a significant association with an elevated risk of self-harm repetition. Notably, the presence of psychiatric disorders, particularly affective and personality disorders, exhibited a 1.54-fold and 2-fold increase, respectively, in the likelihood of recurrence. Additionally, lifetime psychiatric history was associated with a 1.77-fold increase, while history of non-suicidal self-injuries showed a 1.79-fold increase in the likelihood of self-harm repetition. Regarding the self-harm method of the index episode, there was a trend (*p* = .066), with cutting or piercing showing a substantial 2.57-fold increase (Fig. 3 displays the proportion of recurring episodes as a function of each significant multivariable predictor of repetition).

## Discussion

In this study, we investigated the differences in characteristics, patterns, and temporal trends of self-harm episodes among young people over a five-year follow-up period, both before and after the COVID-19 period, between 2018 and 2022. Three main results were found. Firstly, our findings revealed a notable increase in the rate of first registered self-harm episodes within the” post-COVID-19 period” despite similar observation duration in both periods. Specifically, these cases peaked between December 2020 and March 2021, with a monthly increase of up to 23.31% followed by a gradual decline, approaching initial rates. Secondly, patterns of self-harm during the COVID-19 periods differed by age, lifetime substance use, type of index episode and recurring episodes within 30 days of the first attempt. Thirdly, an increased risk of recurring self-harm episodes was related to psychiatric disorders (particularly affective and personality disorders), previous psychiatric history, lifetime history of NSSI, and cutting or piercing as a method.

### Increased Self-Harm Episodes Post-COVID-19

Research on self-harm during the COVID-19 pandemic has yielded mixed findings, largely dependent on the observation period. Our study revealed a significant increase in first self-harm episodes during the “post-COVID-19 period”, with a notable peak between December 2020 and March 2021. This finding aligns with previous research reporting increased emergency visits for suicidal behavior among young people during early 2021, approximately one year after the initial COVID-19 outbreak (García-Fernández et al., 2023; Hertz & Barrios, 2021; Mayne et al., 2021).

However, it’s important to note that our findings contrast with some earlier studies. Research conducted at the pandemic onset, with shorter follow-up periods, often report no significant changes in self-harm compared to previous periods (Davico et al., 2021; Isumi et al., 2020; Mourouvaye et al., 2021). Studies with longer observation periods have consistently reported a rise in the number of young people seeking urgent psychiatric care due to suicidal thoughts or behavior (Bersia et al., 2022; Carison et al., 2022; Chadi et al., 2021; Cousien et al., 2021; García-Fernández et al., 2023; Iob et al., 2020; Kirič et al., 2022; Sara et al., 2023).

The increase in self-harm episodes could be attributed to a complex interplay of factors. While pandemic-related stressors, such as restrictions imposed during January-February 2021 after the increase in COVID-19 cases (Sara et al., 2023) and the cumulative effect of prolonged exposure to pandemic-related stressor (Loades et al., 2020) likely played a significant role, other elements warrant consideration. Changes in healthcare-seeking behavior during the pandemic may have influenced reporting patterns, with initial reluctance to visit hospitals potentially suppressing early figures, followed by a subsequent surge (Villaseñor et al., 2023). Additionally, in the post-pandemic period, the heightened awareness among clinicians of suicidal behavior could have led to more sensitive detection and recording of cases. Broader societal shifts, including increased mental health awareness and changes in social media use, may have contributed to the observed trends independently of the pandemic (Corredor-Waldron & Currie, 2024). It is also crucial to consider that the pandemic may have exacerbated pre-existing upward trends in self-harm rates rather than being their sole cause.

Furthermore, it is noteworthy how rates decreased from March 2021 rates start to gradually decrease towards the initial rates. It could be argued, that normalization of social interactions and reducing acute stressors associated such as isolation and disrupted routines may contribute to enhanced psychological well-being (Hawton et al., 2021). However, there is growing evidence of long-lasting mental health impacts after recovering from COVID-19 (Llach & Vieta, 2021). Specifically, further research is required to ascertain whether the increasing trend of suicides in young people will abate once the impact of the pandemic has passed and return to pre-pandemic levels, or whether the social repercussions of the pandemic and the associated containment measures have had an impact on the generation of young people affected by these measures.

Interestingly, while we found an increase in new cases of first self-harm episodes, this did not correlate with an increase in recurring self-harm episodes in our emergency department. This finding warrants further investigation into the factors influencing initial versus recurring self-harm behaviors during the pandemic.

### Changes in Self-Harm Patterns during the Pandemic

Our analysis of the COVID-19 pandemic periods revealed significant changes in self-harm patterns among young people. Those presenting their first self-harm episode were younger (10–14 years), had a lifetime substance use, showed more frequent self-harm episodes with suicidal intention, and displayed a tendency for more rapid reattempts.

This age difference is in line with previous research showing an increasing trend in self-harm presentations among individuals under 16 years during the pandemic (Paterson et al., 2023). Moreover, age has been found to play a significant role in how stress due to pandemic affects emotional well-being (Auger et al., 2023; Barber & Kim, 2021). According to previous research, several factors might contribute to these changes. School closures, quarantine measures and the lack of normal social interactions with peers have led to isolation and problems in developing essential social skills, resulting in increased mental health issues, such as anxiety and depression (Singh et al., 2020). Additionally, back-to-school academic stress after the lockdown period (Bersia et al., 2022; García-Fernández et al., 2023; Gracia et al., 2021) and the influence of social networks during the pandemic on suicidal behavior (Kirič et al., 2022) have been related to an increase of self-harm rates.

Our findings align with previous research establishing a relationship between substance abuse and self-harm in adolescents (Moran et al., 2015; Wilcox, 2004). We found that lifetime drug use was a significant factor in the increased self-harm rates following the pandemic onset. Many youths who have difficulties coping with their problems seek relief from drugs and alcohol (Mars et al., 2019). However, given the exceptional circumstances of the pandemic, further investigation is needed to clarify the causal relationship between drug use and increased self-harm episodes.

Regarding the type of index episode, we found an increase in suicidal attempts versus NSSI during the “post-COVID-19 period”. Thus, our finding is in agreement with previous literature which found that people who have had attempts were significantly more likely to have experienced stressful life events compared to subjects exhibiting other manifestations of self-harm behavior (King et al., 2001). The COVID-19 pandemic emerges as an important stress factor in this context.

### Risk Factors for Recurring Self-Harm Episodes

Our study found that individuals who have committed self-harmed in the “post-COVID-19 period” engaged in subsequent self-harm episodes more rapidly within the next 30 days compared to those in pre-pandemic times. This finding support previous research indicating that shorter time intervals between self-harm episodes increase the risk of subsequent suicidal behavior (Bostwick et al., 2016; Hawton et al., 2003; Olfson et al., 2017). Specifically, the risk of suicide mortality is notably elevated for up to six months following a self-harm episode, peaking one month after the attempt (Geulayov et al., 2019).

Over a five-year follow-up, 35% of our sample exhibit recurrent suicide attempts after an initial episode. These findings align with previous studies identifying prior attempts as the main risk factor of suicidal behavior, with risk increasing with each subsequent attempt (de la Torre-Luque et al., 2023; Gibb et al., 2005; Ribeiro et al., 2016). Adolescents, in particular, show higher recurrence rates (Castellví et al., 2017; Mars et al., 2019; Plener et al., 2015).,

Our results are also in line with previous studies, which revealed that the risk of recurring episodes rises with the frequency of repetitive NSSI episodes in both young individuals (Hawton et al., 2012a, b) and across all age groups (Perry et al., 2012). Similarly to previous research we found other risk factors for recurring self-harm as previous psychiatric history (Brager-Larsen et al., 2022; de Beurs et al., 2019; Ezquerra et al., 2023; Monnin et al., 2012) and a psychiatric diagnosis, particularly affective (Webb, 2002) and personality disorders (Olfson et al., 2017).

Regarding the method used, our results are consistent with studies finding a higher risk of attempt repetition when cutting or piercing was featured (Bennardi et al., 2016; Blasco-Fontecilla et al., 2016; de la Torre-Luque et al., 2023; Hawton et al., 2012a, b). Previous studies suggest that self-harm, such as cutting or piercing, may be addictive (Blasco-Fontecilla et al., 2016), leading individuals to repeat it and potentially experience more episodes.

We failed to find age as a significant risk factor for new episodes, although it is important to bear in mind that our sample consisted of individuals aged between 10 and 25 years. Previous evidence has shown that the relationship between age and self-harm repetition in adolescents is uncertain, and thus further research is required (Rahman et al., 2021).

### Clinical and Research Implications

Our results have significant implications for clinical practice and public health policy. The findings underscore the need for targeted interventions and enhanced mental health support for young people, particularly those aged 10–14, in the post-pandemic period. Clinicians should be alert to the increased risk of self-harm and implement early detection and rapid intervention strategies, especially for individuals with a history of psychiatric disorders or substance use. From a research perspective, longitudinal studies are crucial to understand the long-term mental health consequences of the pandemic on adolescents and young adults. In this context, governments should prioritize comprehensive mental health strategies with a strong emphasis on prevention and early intervention. These strategies should include improving access to mental health care, making interventions more affordable and timelier, and implementing public health campaigns to promote mental wellbeing and reduce stigma. Additionally, governments should invest in digital mental health services and foster collaboration between healthcare providers and community organizations to create integrated support systems, thereby strengthening overall community resilience in the face of ongoing and future crises.

### Strengths and Limitations

This study has several strengths, including an extensive sample that focuses on individuals between the ages of 10 and 25 years who are particularly vulnerable to self-harm, a longitudinal design, and an extended assessment period of five years that ended in December 2022. However, it also has some limitations that should be noted. Firstly, our study is based on the review of electronic records, and no structured interview or measures were carried out. Secondly, we were not able to capture data on people who sought help from other services and hospitals different than the UHVR. In addition, most young people with suicidal behaviors, specially non-suicidal self-harm, do not seek formal professional assistance; rather, they are more inclined to seek informal support from their peers (Michelmore & Hindley, 2012). This may thus have slightly underestimated the rate of index episodes and their repetition. Thirdly, there may be several other factors influencing self-harm that were not included in the current study, such as culture, cyberbullying, genetic factors, medication, and participants’ socioeconomic level. Fourthly, although our study has a five-year follow-up period, longer studies are needed to better understand the long-term adverse consequences of the pandemic on adolescents’ and young adults’ mental health, especially the impact on suicidal behavior, including self-harm. Finally, our sample predominantly reflects a Hispanic cultural identity, which presents specific characteristics that could influence the results. In particular, social and economic factors, such as access to healthcare, may affect our findings and recommendations. As a result, it may be challenging to generalize these results to other cultures or ethnicities.

## Conclusions

Our study found an increase in self-harm among adolescents and young adults in the post-COVID-19 period, especially between December 2020 and March 2021, followed by a decreased after that. Those who self-harmed for the first time in this period tended to be younger, with a history of substance use, and an index event characterized by attempting suicide over NSSI, along with more rapid recurring episodes. It is crucial to implement social and public health policies to prevent suicide during pandemics and crises and promote mental health and youth well-being. Additionally, our study highlights the importance of considering lifetime history of self-harm, lifetime psychiatric history and psychiatric diagnosis, such as affective and personality disorders, which might help clinicians to identify which adolescents and young people are at greater risk of self-harm repetition in the future.

## Electronic Supplementary Material

Below is the link to the electronic supplementary material.

Supplementary Material 1

## Data Availability

Data available under requirement.
