# Supplementary material for: Characteristics of Self-Harm in an Emergency Situation Among Youth: A Longitudinal Five- Year Cohort Study
Source: Res Child Adolesc Psychopathol. 2025 Apr 26;53(8):1185–98. doi: 10.1007/s10802-025-01309-x (PMC12357799; doi:10.1007/s10802-025-01309-x)
Supplement: Supplementary file 1 — Supplementary Material 1 [file 10802_2025_1309_MOESM1_ESM.docx]

**Supplementary Materials**

| **Segment** | **Lower Endpoint** | **Upper Endpoint** | **Monthly Percent Change (MPC)** | **Lower CI** | **Upper CI** |
| --- | --- | --- | --- | --- | --- |
| 1 | January 2018 | Decebember 2020 | -0.0155 | -3.8051 | 1.3905 |
| 2 | Decebember  2020 | March 2021 | 23.3137* | 0.2242 | 35.6839 |
| 3 | March 2021 | Decebember  2022 | -2.4339 | -6.068 | 0.528 |

| Index | Month | n | Observed Crude Rate | Standard Error | Modeled Crude Rate | Jointpoint Location | Monthly Percent Change |
| --- | --- | --- | --- | --- | --- | --- | --- |
| 1 | 01/01/2018 | 11 | 2.49 | 0.75 | 2.42 |  | -0.0155 |
| 2 | 01/02/2018 | 10 | 2.26 | 0.7151 | 2.42 |  | -0.0155 |
| 3 | 01/03/2018 | 8 | 1.81 | 0.6396 | 2.42 |  | -0.0155 |
| 4 | 01/04/2018 | 3 | 0.68 | 0.3917 | 2.42 |  | -0.0155 |
| 5 | 01/05/2018 | 9 | 2.04 | 0.6784 | 2.41 |  | -0.0155 |
| 6 | 01/06/2018 | 13 | 2.94 | 0.8153 | 2.41 |  | -0.0155 |
| 7 | 01/07/2018 | 8 | 1.81 | 0.6396 | 2.41 |  | -0.0155 |
| 8 | 01/08/2018 | 10 | 2.26 | 0.7151 | 2.41 |  | -0.0155 |
| 9 | 01/09/2018 | 13 | 2.94 | 0.8153 | 2.41 |  | -0.0155 |
| 10 | 01/10/2018 | 6 | 1.36 | 0.5539 | 2.41 |  | -0.0155 |
| 11 | 01/11/2018 | 11 | 2.49 | 0.75 | 2.41 |  | -0.0155 |
| 12 | 01/12/2018 | 10 | 2.26 | 0.7151 | 2.41 |  | -0.0155 |
| 13 | 01/01/2019 | 15 | 3.39 | 0.8758 | 2.41 |  | -0.0155 |
| 14 | 01/02/2019 | 14 | 3.17 | 0.8461 | 2.41 |  | -0.0155 |
| 15 | 01/03/2019 | 9 | 2.04 | 0.6784 | 2.41 |  | -0.0155 |
| 16 | 01/04/2019 | 10 | 2.26 | 0.7151 | 2.41 |  | -0.0155 |
| 17 | 01/05/2019 | 10 | 2.26 | 0.7151 | 2.41 |  | -0.0155 |
| 18 | 01/06/2019 | 15 | 3.39 | 0.8758 | 2.41 |  | -0.0155 |
| 19 | 01/07/2019 | 7 | 1.58 | 0.5983 | 2.41 |  | -0.0155 |
| 20 | 01/08/2019 | 9 | 2.04 | 0.6784 | 2.41 |  | -0.0155 |
| 21 | 01/09/2019 | 15 | 3.39 | 0.8758 | 2.41 |  | -0.0155 |
| 22 | 01/10/2019 | 8 | 1.81 | 0.6396 | 2.41 |  | -0.0155 |
| 23 | 01/11/2019 | 12 | 2.71 | 0.7834 | 2.41 |  | -0.0155 |
| 24 | 01/12/2019 | 5 | 1.13 | 0.5057 | 2.41 |  | -0.0155 |
| 25 | 01/01/2020 | 16 | 3.62 | 0.9045 | 2.41 |  | -0.0155 |
| 26 | 01/02/2020 | 17 | 3.84 | 0.9324 | 2.41 |  | -0.0155 |
| 27 | 01/03/2020 | 8 | 1.81 | 0.6396 | 2.41 |  | -0.0155 |
| 28 | 01/04/2020 | 13 | 2.94 | 0.8153 | 2.41 |  | -0.0155 |
| 29 | 01/05/2020 | 7 | 1.58 | 0.5983 | 2.41 |  | -0.0155 |
| 30 | 01/06/2020 | 12 | 2.71 | 0.7834 | 2.41 |  | -0.0155 |
| 31 | 01/07/2020 | 10 | 2.26 | 0.7151 | 2.41 |  | -0.0155 |
| 32 | 01/08/2020 | 9 | 2.04 | 0.6784 | 2.4 |  | -0.0155 |
| 33 | 01/09/2020 | 6 | 1.36 | 0.5539 | 2.4 |  | -0.0155 |
| 34 | 01/10/2020 | 8 | 1.81 | 0.6396 | 2.4 |  | -0.0155 |
| 35 | 01/11/2020 | 6 | 1.36 | 0.5539 | 2.4 |  | -0.0155 |
| 36 | 01/12/2020 | 12 | 2.71 | 0.7834 | 2.4 | Joinpoint 1 |  |
| 37 | 01/01/2021 | 8 | 1.81 | 0.6396 | 2.96 |  | 23.3137* |
| 38 | 01/02/2021 | 20 | 4.52 | 1.0113 | 3.65 |  | 23.3137* |
| 39 | 01/03/2021 | 17 | 3.84 | 0.9324 | 4.51 | Joinpoint 2 |  |
| 40 | 01/04/2021 | 20 | 4.52 | 1.0113 | 4.4 |  | -2.4339 |
| 41 | 01/05/2021 | 25 | 5.65 | 1.1307 | 4.29 |  | -2.4339 |
| 42 | 01/06/2021 | 17 | 3.84 | 0.9324 | 4.19 |  | -2.4339 |
| 43 | 01/07/2021 | 14 | 3.17 | 0.8461 | 4.08 |  | -2.4339 |
| 44 | 01/08/2021 | 14 | 3.17 | 0.8461 | 3.98 |  | -2.4339 |
| 45 | 01/09/2021 | 17 | 3.84 | 0.9324 | 3.89 |  | -2.4339 |
| 46 | 01/10/2021 | 16 | 3.62 | 0.9045 | 3.79 |  | -2.4339 |
| 47 | 01/11/2021 | 10 | 2.26 | 0.7151 | 3.7 |  | -2.4339 |
| 48 | 01/12/2021 | 18 | 4.07 | 0.9594 | 3.61 |  | -2.4339 |
| 49 | 01/01/2022 | 11 | 2.49 | 0.75 | 3.52 |  | -2.4339 |
| 50 | 01/02/2022 | 22 | 4.98 | 1.0607 | 3.44 |  | -2.4339 |
| 51 | 01/03/2022 | 22 | 4.98 | 1.0607 | 3.35 |  | -2.4339 |
| 52 | 01/04/2022 | 12 | 2.71 | 0.7834 | 3.27 |  | -2.4339 |
| 53 | 01/05/2022 | 15 | 3.39 | 0.8758 | 3.19 |  | -2.4339 |
| 54 | 01/06/2022 | 9 | 2.04 | 0.6784 | 3.11 |  | -2.4339 |
| 55 | 01/07/2022 | 14 | 3.17 | 0.8461 | 3.04 |  | -2.4339 |
| 56 | 01/08/2022 | 11 | 2.49 | 0.75 | 2.96 |  | -2.4339 |
| 57 | 01/09/2022 | 10 | 2.26 | 0.7151 | 2.89 |  | -2.4339 |
| 58 | 01/10/2022 | 12 | 2.71 | 0.7834 | 2.82 |  | -2.4339 |
| 59 | 01/11/2022 | 14 | 3.17 | 0.8461 | 2.75 |  | -2.4339 |
| 60 | 01/12/2022 | 12 | 2.71 | 0.7834 | 2.69 |  | -2.4339 |
